# Carbon Monoxide in Healthcare Monitoring Balancing Potential and Challenges in Public Health Perspective: A Narrative Review

**DOI:** 10.7759/cureus.74052

**Published:** 2024-11-19

**Authors:** V Mounika, Indumathi K P, Sibyl Siluvai, Krishnaprakash G

**Affiliations:** 1 Department of Public Health Dentistry, SRM Kattankulathur Dental College and Hospital, SRM Institute of Science and Technology, Kattankulathur, IND

**Keywords:** biomarker, cancer, carbon monoxide, carbon monoxide releasing molecules, cardiovascular illness, nonalcoholic fatty liver disease (nafld)

## Abstract

Carbon monoxide (CO) has medicinal potential and harmful qualities. However, excessive exposure to CO can lead to severe organ failure. CO is exogenously and endogenously generated within the human body. Ongoing research aims to uncover the beneficial aspects of CO. It serves as a biomarker for inflammation and other serious illnesses. Preclinical trials exploring CO's application have indicated potential benefits in addressing conditions such as Ischemia, Tendonitis, Neuropathic pain, and even cancer therapy. Cardiovascular disease emerges as a particularly promising target for CO therapy due to its potent vasodilatory effects. While research into CO-based therapeutics has shown promise in experimental and preclinical settings, clinical translation and widespread adoption remain in the early stages. This review will illuminate the advantageous role of CO as a biomarker alongside the obstacles and challenges associated with its implementation.

## Introduction and background

Carbon monoxide (CO) is one of the most dangerous gases. This gas can be lethal during prolonged exposure. A high degree of suspicion, a wintertime clustering of patients, and a comprehensive history all contribute to the diagnosis. High CO concentration in breathing affects the heart and brain, among other important organs. Therefore, it makes sense why it is called a "silent killer" [1]. However, scientists are investigating ways to use CO's beneficial attributes at low doses to treat illnesses. The novel advances in technology and medicine suggest how it may be used in a therapy plan. Controlled exposure to low doses of CO, also known as CO treatment, has shown potential therapeutic effects in organ transplantation and ischemic injuries due to its ability to activate protective mechanisms in cells. It assesses the function of CO in the human body, its origins, and its potential for use in disease diagnosis, considering sensor technology, standards, moral considerations, and legal frameworks.

In medical contexts, the danger of unintentional CO overexposure during therapy is a source of worry. Improperly calibrated equipment, insufficient ventilation, or inappropriate dosage techniques might result in excessive CO administration, raising the risk of acute toxicity and jeopardizing patient safety. CO poisoning directly caused over 2,000 suicides and over 500 accidental deaths, according to the National Health Care Survey conducted in 2005 by the Centres for Disease Control and Prevention [2]. With significant care and coordination, a massive preschool poisoning with CO has prevented serious casualty occurrences endangering children and workers [3].

CO has therapeutic benefits, such as anti-inflammatory and cytoprotective properties, which may be useful in treating inflammatory illnesses and organ transplant problems. CO medicinal benefits remain underutilized because of restricted resources, information gaps, and a scarcity of clinical studies. Expanding research on CO-based therapeutics might lead to major healthcare advantages, disputing its image as a harmful toxin. This literature review discusses CO's potential in healthcare, emphasizing its beneficial effects when used in controlled, low doses despite its toxic nature.

## Review

Methodology

A comprehensive search was undertaken using Pubmed, Scopus, Web of Science, websites (epa.gov, cpsc.gov, verywellhealth.com), newsletters (Medical News Today, Science Daily), and books (Gas Biology Research in Clinical Practice by Karger, Carbon Monoxide in Drug Discovery by Wiley) to conduct this review. Keywords employed included carbon monoxide in healthcare, biomarker, carbon monoxide poisoning, toxicology, monitoring carbon monoxide levels, health effects of carbon monoxide, public health impact carbon monoxide, and carbon monoxide exposure. Owing to the limited availability of research related to the subject of interest, existing literature between 1949 and 2024 was included in the review. Search results were improved by reading the bibliographies of the articles found. It ensured the topic of CO in healthcare monitoring was thoroughly explored.

*Inclusion* *Criteria*

1) Research on CO in healthcare professions, CO toxicity, clinical trials of CO as medicine

2) Articles discussing the public health impact of CO exposure

3) Studies focusing on both clinical and non-clinical healthcare settings (home care, emergency medicine, hospitals) 

4) Original research, meta-analyses, books, newsletters, and .gov websites for statistics

5) English-language publications

6) Studies addressing the balance of benefits and challenges of CO monitoring in healthcare

Exclusion Criteria

1) Studies focusing on industrial settings not related to healthcare

2) Case reports with insufficient data for generalization

3) Research unrelated to monitoring and healthcare (purely toxicology studies without relevance to healthcare monitoring)

4) Non-peer-reviewed sources (conference abstracts, opinion pieces)

Physiological role of carbon monoxide

The preponderance of endogenous CO is produced through a reaction catalyzed by the enzyme heme oxygenase (HO). There are three isoforms of HO. Inducible HO (HO-1) is recognized for its function in heme oxidation and the resulting formation of CO and biliverdin. Constitutive HO (HO-2) contributes to CO generation and heme oxidation. Little is known about the biological purpose of HO-3 [4]. A limited amount of CO arises from the breakdown of heme derived from other hemoproteins, such as cytochrome P450. The gut microbiota may also produce CO [5,6]. The involvement of endogenous CO generation through the skin may lead to novel therapeutic techniques. Skin may play an unexpected role in CO dynamics, revealing the complex interplay between light, chemicals, and our bodies [7]. Skin gas is an intricate combination of organic and inorganic volatile chemicals emerging from the skin across dermal glands, circulation, and epidermal reactions. CO is one of the constituents that is eliminated dermally [8].

Sources of carbon monoxide exposure

CO exposure can occur outdoors or indoors, such as at work or home. Certain professions, such as mining and welding, carry a significant risk of CO exposure. Since paint remover contains dichloromethane, it would be interesting to know if paint strippers also produce CO during metabolism [9,10]. Among outdoor exposure, vehicle emissions and factory operations are the main sources of CO emissions. Smoking is unquestionably harmful to one's health. The rate of inhalation of tobacco smoke is greater outdoors than indoors [11]. It is impossible to ignore the negative effects of smoking on the heart, lungs, and overall health. It can inflict harm both indoors and outdoors. Teenagers are urged to make it a habit. Passive smokers are more likely to get heart disease, strokes, respiratory infections, and other illnesses. Third-hand smoke is another issue. It consists of particles and gases left over from smoking. These compounds can be placed on surfaces and then released as gases into the atmosphere. There is still concern about third-hand smoke, especially among small infants who may come into contact with contaminated surfaces [12].

Carbon monoxide as a biomarker in healthcare

**Figure 1:**
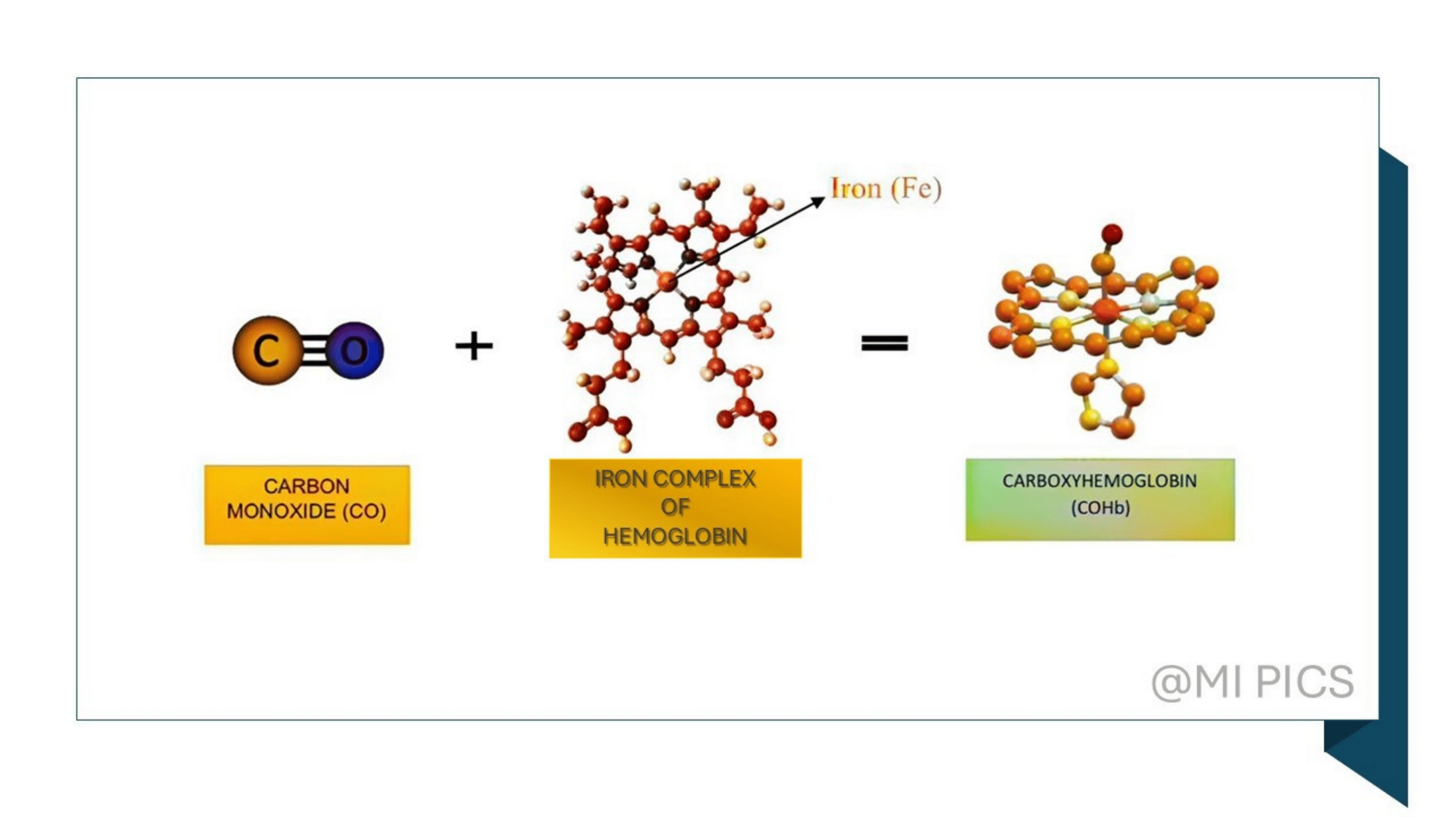
Structural formation of COHb Fe: Iron; CO: Carbon monoxide; COHb: Carboxyhemoglobin

A diagnostic biomarker for determining brief exposure to CO is exhaled CO (exhCO). The exact values have been assessed as possible markers of inflammation in several illnesses such as cystic fibrosis, asthma, pulmonary cancer, chronic obstructive pulmonary disease (COPD), and during critical care or surgery. With the help of exhCO, smoking status can also be assessed [13]. Carboxyhemoglobin (COHb) is produced when CO and the iron atom complex of hemoglobin (Hb) form a strong coordination link (Figure 1). One special biomarker for determining a person's exposure to CO is COHb.

Researchers suggest using an airtight gas syringe and gas chromatography-mass spectrometry (AGS-GC-MS) to measure the total amount of CO in the blood (TBCO). The range of TBCO values is 1.63 -104 nmol/mL of headspace is comparable to 0.65-41.6 μmol/mL of blood plasma. This technique detects higher levels of dissolved CO than previously thought, which may help to explain symptom inconsistencies and decrease incorrect diagnosis [14]. CO can be used significantly in healthcare if used in an appropriate amount. Based on the recently discovered biological benefits of CO, some clinical studies have been conducted to examine the impact of exogenous CO, either through CO-releasing molecules (CO-RMs) or inhalation. It includes human mitochondrial synthesis preconditioning in aortic valve replacement procedure patients, pulmonary inflammatory reactions after pyrogen administration, reducing post-operative ileus following colon resection to treat sepsis-induced acute respiratory distress syndrome, addressing chronic inflammation in COPD, and enhancing organ tolerance in kidney transplant recipients. Cardiovascular illness may be the most promising target for CO therapy. However, several processes underlying the therapeutic effects of CO on the heart tissue have to be explored [15].

Opportunities in healthcare

**Figure 2:**
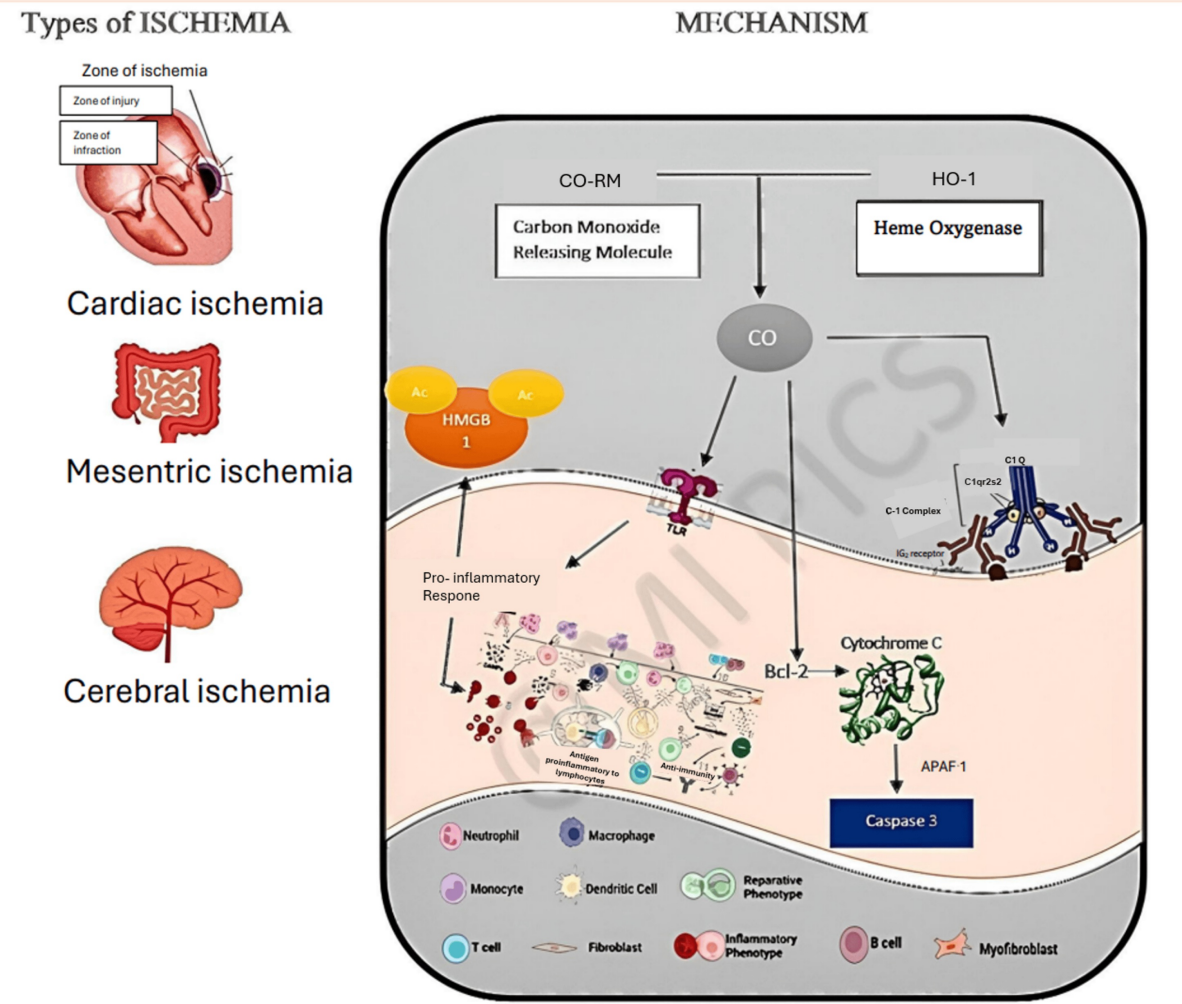
CO in treating ischemia CO: Carbon monoxide; Bcl-2: B-cell lymphoma 2; APAF 1: Apoptotic protease activating factor 1; C-1 complex: Complement component 1 (protein complex); C1Q: Complement component Q-1; IG2: Immunoglobin-2; TLR: Toll-like receptor; HMGB 1: High mobility group box 1; Ac: Acidic C-terminal domain

Since its discovery in 1949, CO has been the focal point of scientific scrutiny and research, especially after establishing CO is also produced internally, notably in situations involving increased erythrocyte breakdown [16,17]. CO appears to take on the effect of endogenous HO-1 in protecting cardiomyocytes from ischemia-reperfusion damage [18]. Revascularization treatment should be initiated as soon as possible after a cardiac arrest to limit myocardial damage. Within the myocardium, the increase in inducible HO-1 following ischemia-reperfusion insult has been highlighted as an indispensable guardian of heart protection, potentially functioning not only by CO generation but also by metabolizing cytotoxic heme [19,20]. Nevertheless, ischemia-reperfusion damage is brought on by the ischemic heart's oxygen replenishment [21]. When the cardiovascular system experiences ischemia load or a persistent increase in intraventricular pressure, all encapsulated cells, even cardiac cells, generate pro-inflammatory cytokines in reaction to damage [13]. Exogenous CO administration can imitate HO-1 initiation, reduce cellular suicide, decrease infarct size, and promote contractility in the rat heart [21-23]. CO has considerable promise for reducing ischemia/reperfusion damage (Figure 2) [24].

CO is also useful in heart transplants. Its anti-proliferative effect on smooth cells reduces organ rejection and increases organ survival by stimulating the recipient's immunological response [15]. CO suppresses the development of smooth muscle cells, both directly and indirectly, decreasing atherosclerosis genesis [25]. So far, the ingestion of CO in anticipation of these favorable effects on heart health has been implemented via a delivery device for inhaled gas or by a medicine with CO-RMs [26].

In liver transplants, CO has been shown to improve survival rates by reducing ischemia-reperfusion injury, which occurs when blood flow resumes to the tissue after a period of oxygen deprivation. Additionally, CO treatment may support liver regeneration following damage, positioning it as a potential therapeutic agent for conditions such as cirrhosis and fibrosis. Research indicates the regulated administration of CO can mitigate inflammation and oxidative stress, both significant contributors to hepatic damage. CO facilitates anti-inflammatory mechanisms via pathways such as HO-1, promoting cellular viability and diminishing tissue injury in the liver.

Musculoskeletal disorders now pose a significant socioeconomic cost. Rotator cuff tears (RCTs) and rotator cuff disease (RCDs) are the two most common and crippling, with prevalence rates ranging from 30 to 70% of the adult population [27,28]. Thus, enhancing tendon healing following injury may be possible with a targeted CA9/12 inhibition. The study, approved by the Massachusetts General Hospital’s Institutional Review Board and followed Principles of Laboratory Animal Care, found CO inhalation for 4.5 and 7.5 hours can repair muscle damage induced by ischemia-reperfusion in a hind limb model in mice [29]. Mice treated with CO showed substantial reductions in serum and tissue cytokines (p<0.05) [29]. CO was additionally examined for its ability to improve recovery after tendon damage. Its ability to regulate inflammation implies it may be useful in promoting tendon recovery, which is essential for muscle repair. CO, an endogenous naturally occurring gas transmitter promoting anti-oxidative, anti-apoptotic, and anti-inflammatory routes, may have the ability to modify the onset of tendinitis when supplied safely and regulated [30]. As it has been demonstrated that CO plays a significant role in regulating inflammation, substances known as CO-RMs may be appropriate [28].

Cancer refers to illnesses defined by uncontrolled cell proliferation and metastasis development [31]. Cancer treatment can be in a variety of ways depending on the cancer type and stage. Typical treatments include surgery to remove the tumor, medications to kill or restrict the development of cancer cells (chemotherapy), and radiation therapy to target and eliminate cancer cells. The standard difference is that immunotherapy easily allows the body to fight against cancer using its immune system rather than a targeted treatment that determines the striking of specific chemicals involved in the growth and proliferation of cancer. Recent advancements in cancer therapy are CO-mediated gas therapy, combined use of CO chemotherapy, photothermal therapy (PTT), photodynamic therapy (PDT), and immunotherapy [32].

CO-RMs can prevent the formation of new blood vessels to nourish cancer tissues, induce cancer cell apoptosis, and impair the tumor microenvironment to inhibit oncogenic growth. Finally, CO-RMs can sensitize cancer cells to other therapies such as radiation and chemotherapy making these treatments more effective. Through personalized delivery, CO is delivered to maximum medicinal potential and minimum side effect impact. A potential method is to restrict or terminate the excessive multiplication of cancer cells, such as by inducing cell cycle arrest [33]. A new research investigation using breast cancer cell models discovered that administering therapy with four readily accessible CO-RMs including CO-RM-1, CO-RM-2, CO-RM-3, and CO-RM-A1, significantly reduced the levels of excreted VEGF in these breast cancer cells, with CO-RM-2 showing the highest efficiency followed by CO-RM-3 [34].

The study of CO-RMs as pro-drugs was able to deliver CO to cells and tissues. This demonstrated a promised CO-based pharmaceutical method. [35]. In recent years, the physiological governing CO function in the cancer process has been increasingly revealed, and CO-related Nano-drugs have been investigated. It demonstrates improved application possibilities in cancer therapy and introduces novel therapeutic concepts [32].

Hepatic lipid buildup is frequently related to ongoing inflammatory conditions. CO has anti-inflammatory properties, inhibiting the synthesis of cytokines causing inflammation, including tumor necrosis factor (TNF-α) and interleukin-6 (IL-6), which can lead to liver impairment and fat storage. Non-alcoholic fatty liver disease (NAFLD) is a condition where fat builds up in the livers of people who consume little or no alcohol.

From simple fatty liver (steatosis) to more severe conditions such as non-alcoholic steatohepatitis (NASH), a condition that can lead to liver inflammation, scarring, cirrhosis, or cancer. NAFLD is linked tightly to obesity, insulin resistance, type 2 diabetes, and metabolic syndrome. It is usually asymptomatic and found incidentally on blood testing or imaging. Catching the disorder early on is extremely important to keep progression from happening. CO stimulates sestrin-2 expression and mitigates hepatic steatosis in NAFLD produced by a methionine/choline-deficient diet [36]. CO-induced protection against cell death necessitates both NO generation and HO-1 activity, demonstrating for the first time a crucial synergy between these chemicals that confers significant cytoprotection [37]. CO causes a spike in the sestrin-2 phase, which helps promote phagocytosis [36].

Neuropathy is a clinical symptomatology marked by dysesthesia and hyperalgesia that is challenging to cure, even with the most effective analgesics. This disorder is caused by the activation of spinal microglia, changes in the plasticity of cerebral nociceptive pathways, and regional responses to inflammatory agents [38,39]. CO produced by HO-1 has antinociceptive properties during inflammation, but its significance during neuropathic pain is uncertain [40]. An investigation has demonstrated CO significantly reduces neuropathic pain in mice. After sciatic nerve injury, there is an exchange between CO and the nitric oxide (NO) systems that indicates that enhancing the endogenous (cobalt protoporphyrin (CoPP)), a well-known heme oxygenase 1 inducer, or exogenous (CO-RMs) production of CO could be a novel way to treat neuropathic pain [40]. Even one injection via the abdomen of CO-RM-2 demonstrated anti-allodynic efficacy and boosted buprenorphine/ morphine analgesia contrasted with the consequences of these medications, fully removing the neural pain sensations [41].

Challenges

One of the main challenges in using CO as therapy is the difficulty of gas standardization. It involves sophisticated technical information, calibration protocols, and regulatory compliance. When considering CO use for treatment, it is essential to recognize its potential benefits and limitations. There are no set norms, processes, or safety concerns. Thus, getting regulatory clearance for CO-based medicines might be difficult. Before authorizing CO therapies for clinical use, regulatory bodies want strong proof of safety, effectiveness, and quality control, which delays the development and regulatory review process.

Prospective clinical trials are necessary to establish efficacy and safety for treating neuropathic pain. CO treatment involves a variety of waves and administration mechanisms. The absence of established norms makes it difficult to execute consistently across various environments [42]. While CO shows promise in managing neuropathic pain, its drawbacks emphasize the need for further research and cautious implementation. Clinicians should carefully evaluate their role in individual cases.

Although this therapeutic gas has intriguing potential, as far as we know, there is a gap for transdermal CO delivery that might be especially helpful for wound healing or swollen tendon treatment [40]. The obvious and risky use of gas containers, similar to gas delivery systems for NO, nitrogen dioxide/oxygen, or xenon, or their lack of use for the safe and local creation of these gases, pose a hurdle to the transdermal administration of gases [40,43].

CO, a gas with numerous properties, performs fascinating roles in cardiovascular functions. It acts as a potent vasodilator, relaxing blood vessels. This property can be beneficial in conditions such as hypertension and ischemic heart disease. However, the challenges remain the same: continuous administration of CO is quite challenging due to its lethal causes. Clinicians should weigh the CO before administration and continuous monitoring is required. Excess ingestion of CO can harm the heart by decreasing the heart’s pumping capacity and impairing function. In addition, chronic smokers are already exposed to elevated levels of CO due to cigarette smoke [44,45]. So, their values might vary.

Obstacles of treatment

CO is poisonous by nature. Significant doses inhaled uncontrollably can cause major neurological disorders and systemic problems. CO therapy offers opportunities as well as difficulties. Let us examine the obstacles related to its therapeutic implementation. Many doctors are actively focusing on minimizing the sickness prevalence and fatalities of individuals experiencing CO overdose, such as reducing the harmful consequences of CO [46,15].

One of the most evident CO problems is that it is intrinsically hazardous due to its affinity for Hb, resulting in COHb and lowering oxygen-carrying capacity. CO treatment presents intrinsic safety hazards, including the possibility of worsening hypoxemia, myocardial instability, dementia, and tissue damage. Ensuring patient safety during CO therapy requires detailed risk assessment, monitoring, and timely management in the event of adverse events. In addition, identifying the correct dose for medicinal purposes is sometimes difficult. Introducing CO-based treatments in hospital settings necessitates specialized infrastructure, equipment, and trained individuals who can properly deliver and monitor CO levels. Providing enough resources and expertise for CO therapy may cause logistical issues, especially in resource-constrained or remote hospital settings. Researchers, healthcare professionals, regulators, and industry stakeholders must work together to address these issues to progress the development, standardization, and use of CO-based medicines while maintaining patient safety and regulatory compliance.

Future direction and research opportunities

CO in healthcare offers an opportunity to lead to novel treatment approaches and scientific advances in the medical field. The future direction can lead to many pharmacological benefits. It is possible to examine further the molecular processes behind CO's positive benefits, including its anti-inflammatory, anti-apoptotic, and vasodilatory capabilities. Identifying specific cellular targets and signaling pathways modulated by CO develops targeted therapies for diseases, including ischemic injury, neurodegenerative disorders, and inflammatory conditions. Controlled CO release is the major obstacle in therapeutic use, which can be solved via nano-formulation to target particular organs or tissues. CO-related nano drugs show potential as cancer treatments. These findings provide new opportunities for revolutionary therapeutics [36].

Another opportunity is investigating CO use in biocompatible forms such as hydrogels or liposomes using nanotechnology. Solvent, light, temperature, and ligand substitution are among the triggers that allow CO-RMs to store and release CO payloads steadily [47]. Utilizing predictive algorithms and biomarkers to determine which patients are most likely to benefit from CO therapy and to tailor treatment plans for optimal safety and effectiveness. Leverage collaborative research projects and well-planned clinical trials to expedite the translation of preclinical results into clinical practice to assess the CO-based treatments' safety, efficacy, and long-term outcomes across varied patient populations and conditions, and conduct thorough clinical investigations. Forward research is essential to completely harness CO’s therapeutic benefits [26]. Despite clinical investigations being infrequent and the number of patients being small, CO remains one of the most promising chemicals with significant promise for the future development of some severe disorders [48].

Public significance

The public relevance of CO as therapy stems from its potential rather than its existing widespread application. Research of CO-based therapeutics has shown promise in experimental models and preclinical investigations, while clinical translation and mainstream adoption are still in the early phases. Since CO gas was previously thought to be a hazardous contaminant, its therapeutic application represents a medical novelty. The anti-inflammatory, cell survival promoter, vascular relaxant, and cytoprotective qualities of CO make it a prospective treatment for disorders such as inflammation, oxidative stress, ischemia-reperfusion injury, and tissue damage. CO therapy, which targets fundamental pathophysiological pathways, may provide new options for illness treatment and improved patient outcomes. CO-based therapeutics have the potential to improve global health by addressing health inequities, increasing access to novel treatments, and decreasing disease burdens worldwide. By expanding research and supporting equitable access to CO-based medicines, the global health community can aim to improve health outcomes for all people.

## Conclusions

Without a doubt, CO is a current topic with advantages and disadvantages with limited information on the benefits yet to be uncovered. CO has potential benefits in treating cardiovascular diseases such as vasodilation, anti-apoptotic activity, immune modulation, and synergy with other gas transmitters. However, CO is inherently toxic with prolonged exposure causing sensory disturbances. Limited evidence supports CO as a treatment and prospective clinical trials are needed to establish its efficacy and safety. Standardization is lacking, making implementation across different settings challenging. Clinicians must weigh CO's potential benefits against risks. Understanding CO's effects and addressing challenges can enhance its therapeutic potential in cardiovascular diseases. While research on CO's medicinal potential continues, it should be used with caution outside of regulated clinical settings. Promoting knowledge, safe practices, and informed decision-making can assist in reducing the hazards associated with CO exposure while encouraging responsible scientific research into its medicinal uses.
